# EcoTILLING Reveals Natural Allelic Variations in Starch Synthesis Key Gene *TaSSIV* and Its Haplotypes Associated with Higher Thousand Grain Weight

**DOI:** 10.3390/genes10040307

**Published:** 2019-04-18

**Authors:** Ahsan Irshad, Huijun Guo, Shunlin Zhang, Jiayu Gu, Linshu Zhao, Yongdun Xie, Hongchun Xiong, Shirong Zhao, Yuping Ding, Youzhi Ma, Luxiang Liu

**Affiliations:** Institute of Crop Sciences, Chinese Academy of Agricultural Sciences/National Engineering Laboratory of Crop Molecular Breeding/National Center of Space Mutagenesis for Crop Improvement, Beijing 100081, China; ahsanirshad@126.com (A.I.); guohuijun@caas.cn (H.G.); zhangshunlin1994@163.com (S.Z.); gujiayu@caas.cn (J.G.); zhaolinshu@caas.cn (L.Z.); xieyongdun@caas.cn (Y.X.); xionghongchun@caas.cn (H.X.); zhaoshirong@caas.cn (S.Z.); dingyuping@caas.cn (Y.D.); mayouzhi@caas.cn (Y.M.)

**Keywords:** starch, *TaSSIV*, EcoTILLING, polymorphism, KASP, haplotype

## Abstract

Wheat is a staple food commodity grown worldwide, and wheat starch is a valuable source of energy and carbon that constitutes 80% of the grain weight. Manipulation of genes involved in starch synthesis significantly affects wheat grain weight and yield. *TaSSIV* plays an important role in starch synthesis and its main function is granule formation. To mine and stack more favorable alleles, single nucleotide polymorphisms (SNPs) of *TaSSIV-A*, *B*, and *D* were investigated across 362 wheat accessions by Ecotype-Targeting Induced Local Lesions IN Genome (EcoTILLING). As a result, a total of 38 SNPs in the amplified regions of three *TaSSIV* genes were identified, of which 10, 15, and 13 were in *TaSSIV-A*, *B*, and *D*, respectively. These 38 SNPs were evaluated by using KASP and six SNPs showed an allele frequency >5% whereas the rest were <5%, i.e., considered to be minor alleles. In the Chinese mini core collection, three haplotypes were detected for *TaSSIV–A* and three for *TaSSIV–B.* The results of an association study in the Chinese mini core collection with thousand grain weight (TGW) and spike length (SPL) showed that *Hap-2-1A* was significantly associated with TGW and *Hap-3-1B* with SPL. Allelic frequency and geographic distribution indicated that the favored haplotype (*Hap-2-1A*) has been positively selected in Chinese wheat breeding. These results suggested that the Kompetitive Allele Specific PCR (KASP) markers can be applied in starch improvement to ultimately improve wheat yield by marker assisted selection in wheat breeding.

## 1. Introduction

Breeding for yield potential of cereal crops due to a surging population is an important priority. Wheat is a staple food commodity for the ever-growing worldwide population. Wheat is central to food security and has a large share of required calories globally. Starch is a valuable source of carbon and energy. In leaves, starch is synthesized during daytime from photosynthetically-fixed carbon and is mobilized in the night time. It is also synthesized in other transient organs such as root cap cells and meristems, but major storage organs are fruits, tubers, seeds, and roots [1]. At the physiological level, seed crop yields are mainly determined by source and sink relationships [2]. Strength of source for photoassimilates is dictated by both the photosynthetic rate and the rate of photoassimilate remobilization from source tissues. In wheat, sink capacity is more important than source accumulation [3], hence, exploitation of enzymes involved in starch synthesis will be more effective in breeding for increased wheat yield [4].

In plants, starch is degraded at night during the process of respiration and helps the formation of sucrose. Starch consists of amylose and amylopectin. Amylopectin is the major part of starch, contributing about 75% of starch granules [5]. Different enzymes are involved in the formation of starch such as ADP-glucose pyrophosphorylases (AGPase) and starch synthases (SS) [6]. SS is controlled by different gene classes such as granule bound starch synthase (GBSS), and starch synthases I, II, III, and IV [5,7]. GBSS completely binds the starch granules and is only responsible for amylose synthesis. SSI, SSII, and SSIII are involved in amylopectin elongation [8], while SSIV controls the number of starch granules in the leaves of *Arabidopsis* [9,10]. The presence of SSIV in thylakoid membranes in *Arabidopsis* suggests that starch granules are initiated at a specific area of the chloroplast. Exon and intron regions of SSIII and SSIV are highly conserved in *Arabidopsis*, rice, and wheat but are different from SSI, SSII, and GBSS [11,12]. The *Arabidopsis* starch mutant plant (*esv1*) cannot synthesize or degrade starch at night and was dwarfed [13]. Overexpression of SSIV increases the level of starch accumulated in the leaves of *Arabidopsis* and potato, which also show higher rates of growth. Overexpression increased starch content in both photosynthetic and sink organs [14,15]. *OsSSIVa* and *OsSSIVb* are mainly expressed in endosperm and leaves, respectively. Starch granule synthesis initiation in rice endosperm does not solely depend on *OsSSIIIa* and *OsSSIVb*, because suppression of these genes did not stop the granule initiation process. These results are different from *Arabidopsis* double mutant in which suppression of these two genes totally stopped granule production [16].

In wheat, *TaSSIV* is closely related to *TaSSIII* according to phylogenetic analysis and these genes share a similar arrangement of exons and introns [7]. The chromosomal location of *TaSSIV* is on the long arm of homoeologous group 1 chromosome and is syntonic with *OsSSIVb* located on chromosome 5 [7]. The presence of *TaSSIII* and *TaSSIV* on the same chromosome but opposite arms and phylogenetic analysis indicates that the evolutionary histories of these genes are related [17]. *TaSSIV-D* mutation of wheat through EMS (ethyl methane sulphonate) showed that the chloroplasts containing 0–2 granules increased significantly while the chloroplasts containing 3–4 granules decreased [18].

EcoTILLING (Ecotype Targeting Induced Local Lesions in Genomes), is an inexpensive, robust, and reliable allele mining approach. This approach is capable of allele mining and identifying genotypes of novel functional and natural variation of known genes [19,20,21,22,23,24,25]. In rice, this technique has been used to characterize five genes of salt tolerance [26]. Similarly, eight genes in tomato related to sucrose synthesis were characterized [27]. In wheat, the EcoTILLLING approach has also been deployed to determine allelic diversity of genes such as kernel hardness genes, *Pin a* and *Pin b* [28]. In the future, new cultivars could be explored by EcoTILLING that contain the alleles originating from their wild relatives.

Single nucleotide polymorphisms (SNPs) have been widely used in marker assisted selection (MAS). SNPs and deletions/insertions (InDels) are nucleotide variations in natural populations that can be significantly associated with agronomic parameters [29]. Comprehensive SNP analysis of genes of particular interest are helpful for association analysis between allelic variations with phenotypic differences and the subsequent MAS of the associated traits [30]. To date, DNA polymorphisms of *TaSSIV* have not been systematically surveyed in wheat. Natural polymorphism can be used for further breeding programmes and to understand the function of *TaSSIV*. There is also a need to know the allelic variation of *TaSSIV* in *A*, *B*, and *D* genomes.

In this study, EcoTILLING was used to detect allelic variation in the targeted region of *TaSSIV*-*A*, *B*, and *D* genomes in 362 wheat accessions collected from diverse geographical areas of China and Pakistan to identify nucleotide diversity. The developed Kompetitive allele-specific PCR (KASP) markers were applied to screen 262 Chinese mini core collection (MCC) and 100 Pakistani wheat accessions for particular nucleotide variation. SNPs were identified with distinguishable haplotypes in the germplasm collection. In addition, association analysis between the haplotypes and phenotypic traits of the MCC was also performed to reveal marker trait associations (MTAs).

## 2. Materials and Methods

### 2.1. Plant Material and DNA Extraction

A panel of 362 wheat accessions was used for EcoTILLING. This panel mainly consisted of 262 mini core collection (MCC) accessions from China and 100 accessions originating from Pakistan (spring wheat) (Table S1). MCC represents 70% of the total wheat genetic diversity available in China [4]. MCC accessions were planted at CAAS Luoyang Experimental Station in Henan Province in 2002 and 2005. Morphological data was collected at the maturity stage, for thousand grain weight (TGW) and spike length (SPL) [31].

### 2.2. DNA Isolation and Molecular Analyses

Genomic DNA (gDNA) was isolated from young seedling leaves using the standard CTAB procedure [32]. Extracted gDNA was checked by using 1% agarose gel electrophoresis for quality and quantified using a NanoDrop ND-1000 spectrophotometer.

### 2.3. Primers Development

The target gene regions with maximum probability for missense variants were predicted using the CODDLE bioinformatics pipeline (http://blocks.fhcrc.org/proweb/). Genomic sequences of the three *TaSSIV* genes were obtained from the GenBank database (http://www.wheatgenome.org/). Multiple alignment of nucleotide sequences was conducted by Bioedit 7.0. Primers were designed using Primer Premier 5.0 (Premier Biosoft International, Palo Alto, CA). Genome-specific primers for *TaSSIV-A* and *TaSSIV-B* were designed on the basis of SNPs and/or InDels; primers were already available for *TaSSIV-D* [18]. Specificity of primers for target genes was tested by PCR amplification in Chinese Spring (CS), Jing411, and nulli-tetrasomic lines of CS. Detailed information for primers is given in Tables S2, S3 and Figure S1.

### 2.4. EcoTILLING

For EcoTILLING, each accession and Chinese Spring (WT) were pooled in a 1:1 ratio after diluting the samples. PCRs were performed in 10 µL final volumes on 100 ng µL^−1^ DNA using 0.4 U/reaction of Taq polymerase (TaKaRa Ex TaqTM). The mixture contained a 4:1 ratio of IRD800-labeled to unlabeled reverse primers and 3:2 ratio of IRD700-labeled to unlabeled forward primers. The EcoTILLING method was followed as described in [33]. After the denaturation step, each sample was loaded on the 6.5% polyacrylamide gel with a 96 well comb. The gel images were analyzed using Gel buddy software and polymorphisms were identified.

### 2.5. Sequencing of Variants

Resultant PCR products were directly sequenced in both directions by Sangon Biotech Co., Ltd., Beijing, China, to confirm the sequence variations. Identified sequence variants were analyzed by the PARSESNP (http://blocks.fhcrc.org/proweb/) and SIFT (Sorting Intolerant from Tolerant) (http://sift.jcvi.org/) programs with default parameters [34].

### 2.6. Development of Functional Markers (FMs) Using KASP

For high-throughput genotyping, KASP primers were developed on the variants of *TaSSIV-A*, *-B*, and *-D* by following standard KASP guidelines (http://www.lgcgenomics.com). The allele-specific primers were developed using the standard FAM (5′ GAAGGTGACCAAGTTCATGCT 3′) and HEX (5′ GAAGGT CGGAGTCAACGGATT 3′) tails with a targeted SNP at the 3′ end. KASP markers were then applied across the entire population. The KASP markers were developed by following standard KASP guidelines [35]. A scatter plot for KASP assays showed clustering of accessions on the *X-(FAM)* and *Y-(HEX)* signals. Detailed information for primers is given in Tables S2, S3 and Figure S1.

### 2.7. Association between SNPs and Agronomic Traits

Descriptive statistics and estimates of variance were conducted using Microsoft Excel 2013. The effect of haplotypes on agronomic traits were analyzed using Student’s *t*-tests at *p* < 0.05 (even 0.01).

## 3. Results

### 3.1. Natural Variation of TaSSIV in Accessions

**Figure 1:**
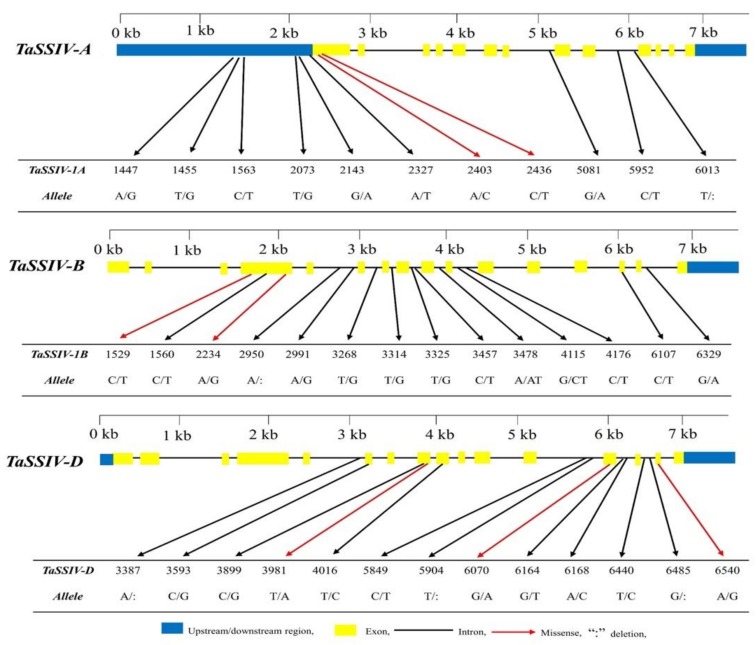
Location of single nucleotide polymorphisms (SNPs) in *TaSSIV-A*, *TaSSIV-B* and *TaSSIV-D*.

Gene structure analysis showed that *TaSSIV-A* consisted of 13 exons and 12 introns while *TaSSIV-B*, and *TaSSIV-D* each consisted of 16 exons and 15 introns. Each gene was exploited in the wheat population for sequence polymorphism by EcoTILLING. A total of 38 putative natural variation sites were identified across the population (Table 1 and Figure 1). Twelve sequence polymorphic sites were identified in the coding region, while 26 were identified in the non-coding region. Out of 38 DNA polymorphisms, 31 were SNPs while seven were InDels. Among the identified sequence polymorphisms, two missense polymorphisms were identified in *TaSSIV-A* and *TaSSIV-B* each, while *TaSSIV-D* possessed three missense polymorphisms. *TaSSIV-B* and *TaSSIV-D* also possessed one and three silent polymorphisms, respectively.

The SIFT computer program predicted that only one sequence polymorphism in *TaSSIV-D* led to an amino acid change (V → I at 800 aa), which was located in the GT-5 domain and may affect the protein function. All other missense polymorphisms of *TaSSIV-A*, *B*, and *D* were present in coiled regions of protein structure and not in functional domains.

### 3.2. KASP Marker Development

**Figure 2:**
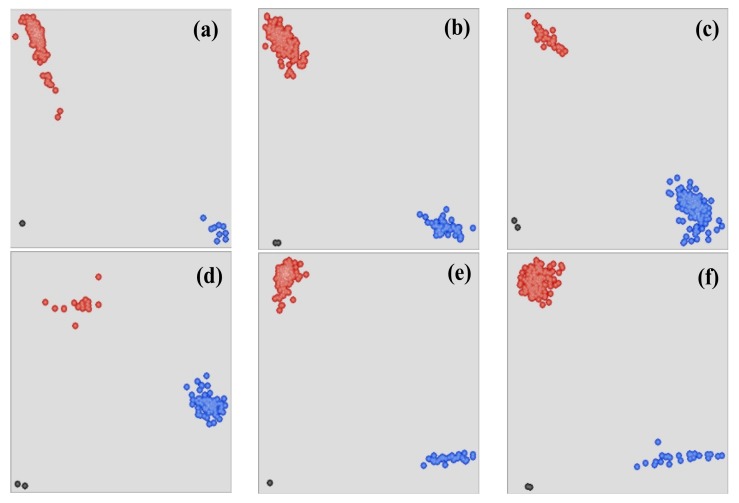
Scatter plot for KASP assays; showing clustering of accessions on the X-(FAM) and Y-(HEX) axes. Accessions colored blue have the FAM- type allele; accessions colored red have the HEX-type allele; black dots represent the NTC (non-template control). (**a**–**d**): *TaSSIV-A.* (**a**) *KASP-A1673T*, (**b**) *KASP-A2403C*, (**c**) *KASP-C2436T*, (**d**) *KASP-C5952T*, (**e**,**f**) *TaSSIV-B.* (**e**) *KASP-C1560T*, (**f**) *KASP-C6107T*.

KASP markers were developed using identified sequence polymorphisms. Among the KASP markers, four markers (*KASP-A1673T*, *KASP-C5952T*, *KASP-A2403C*, and *KASP-C2436T*) in *TaSSIV-A* and two markers (*KASP-C1560T* and *KASP-C6107T*) in *TaSSIV-B* had allele frequencies over 5%. These results were consistent with EcoTILLING. All SNPs of *TaSSIV-D* had allelic frequencies less than 5% and were therefore excluded from further analysis. For KASP-*A11673T, KASP-C5952T, KASP-A2403C*, and *KASP-C2436T* (Figure 2a–d) accessions in blue circles have *A*, *C*, *A*, and *C* alleles while accessions in red circles have *T*, *T*, *C*, and *T* alleles, respectively, for *TaSSIV-A*. Similarly, for *KASP-C1560T* and *KASP-C6107T* (Figure 2e,f) accessions in blue circles have *C* allele whereas accessions in red circles have *T* allele in *TaSSIV-B* gene (Figure 2).

### 3.3. Association Between Haplotypes and Yield-Related Traits

**Figure 3:**
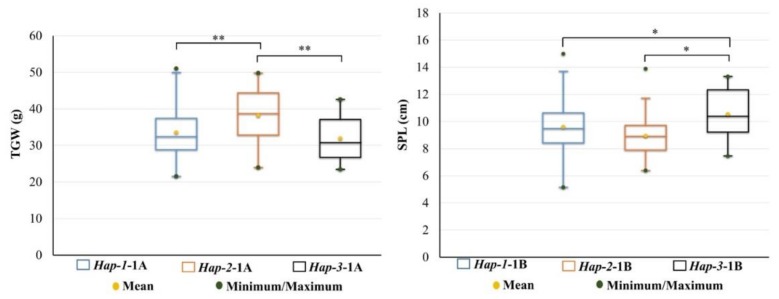
Favorable haplotypes and their interaction with phenotype. TGW, thousand grain weight; SPL, spike length; * *p* < 0.05, ** *p* < 0.01; Two-year data of 2002 and 2005.

There was a total of 14 haplotypes in *TaSSIV-A*. The frequency of each haplotype in MCC was different. Frequencies of the three haplotypes of *TaSSIV-A*, i.e., *Hap-1-1A (AATC)*, *Hap-2-1A (ACTT)*, and *Hap-3-1A* (*ACTC*) were more than 5%. The other eleven haplotypes were less than 5%, marking them as rare haplotypes. Three haplotypes of *TaSSIV-B* i.e., *Hap-1-1B (CC)*, *Hap-2-1B (TC)*, and *Hap-3-1B (CT*) in the wheat population had frequencies more than 5% (Table 2). Association analysis showed that *TaSSIV-A* was associated with TGW; *Hap-2-1A* showed significant difference from the other two haplotypes and had higher TGW. *Hap-3-1B* was the favored haplotype for SPL and was significantly different from *Hap-1-1B* and *Hap-2-1B* (Figure 3). Therefore, these two haplotypes were significantly associated with yield-related traits, which may have potential as functional markers (FMs) to be used in MAS for starch content breeding.

### 3.4. Geographic Distribution of TaSSIV-A and TaSSIV-B Haplotypes

To determine, whether the favored haplotypes for *TaSSIV-A and TaSSIV-B* were selected in wheat breeding, we investigated the geographic distribution of *TaSSIV-A* and *B* in MCC. On the basis of agro-climatic conditions, China has 10 agro-ecological regions, I to X [36].

**Figure 4:**
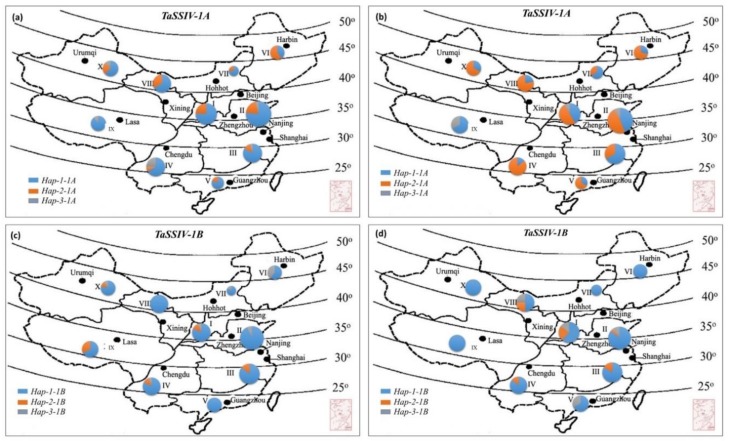
Geographic distribution of *TaSSIV-A and TaSSIV-B* Haplotypes in China. (**a**,**c**) Landraces, (**b**,**d**) modern cultivars. I, Northern winter wheat region; II, Yellow and Huai River valleys winter wheat region; III, Middle and low Yangtze valleys winter wheat region; IV, Southwestern winter wheat region; V, Southern winter wheat region; VI, Northeastern spring wheat region; VII, Northern spring wheat region; VIII, Northwestern spring wheat region; IX, Qinghai-Tibetan spring-winter wheat region; X, Xinjiang winter-spring wheat region. Pie chart size is directly proportional to number of genotypes.

Regions I–IV are the major wheat producing regions based on production and cultivation area. In landraces, the frequency of the favored haplotype *Hap-2-1A* for TGW was low and *Hap-1-1A* was dominant in all major areas. In modern cultivars, the favored haplotype *Hap-2-1A* frequency was high (more than 50%) in regions I–IV (Figure 4). The frequency of *Hap-2-1A* was significantly increased from 25% to 50% in region I, 20% to 48% in region II, 11.67% to 33.37% in region III, and 8% to 85.71% in region IV from landraces to modern cultivars, respectively. The results showed that favored haplotypes experienced positive selection in Chinese wheat breeding programs. Similarly, in *TaSSIV-B* haplotypes, the favored haplotype *Hap-3-1B* was significantly different from *Hap-1-1B* and *Hap-2-1B* for SPL but its frequency was balanced or selectively neutral in landraces and modern wheat cultivars. Therefore, the positive alleles of these haplotypes have the potential to be used in wheat breeding to increase in the grain yield.

**Figure 5:**
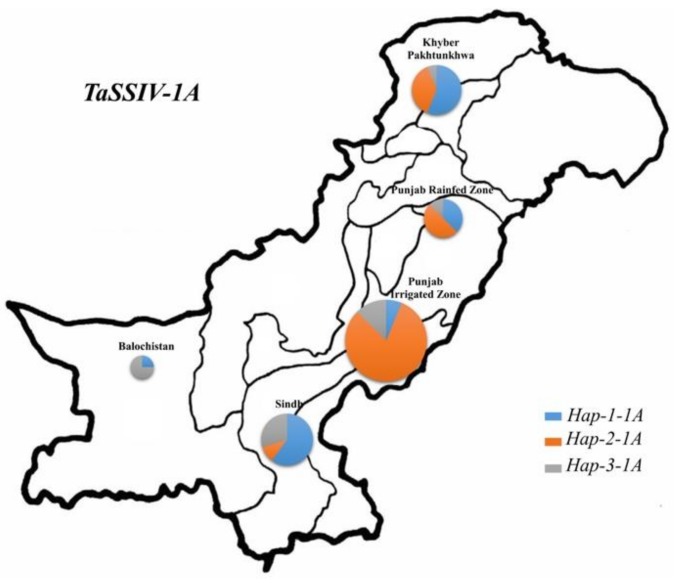
*TaSSIV-A* haplotypes distribution among Pakistani wheat genotypes. I, Punjab rainfed region; II, Punjab irrigated region; III, Sindh region; IV, Balochistan region; V, Khyber Pakhtunkhwa region. Pie chart size is directly proportional to number of genotypes.

In Pakistani wheat accessions, geographic distribution was investigated in *TaSSIV-A* haplotypes. The frequency of *Hap-2-1A* among five different regions of Pakistan was higher in the Punjab irrigated region (78%) and the Punjab rainfed region (52%). The frequency of this haplotype in Khyber Pakhtonkhwa was 35%. (Figure 5).

### 3.5. Positive Selection of Hap-2-1A in China’s Wheat Breeding Process

**Figure 6:**
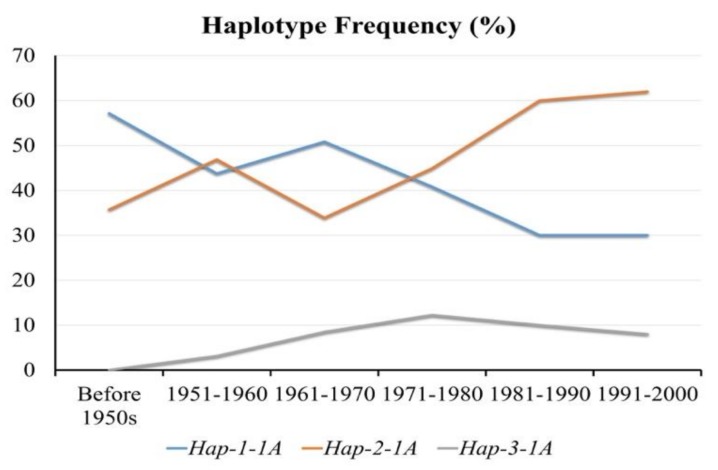
Favored haplotypes of *TaSSIV-A* selected over the history of wheat breeding in China.

On the basis of released time, the wheat population was grouped into six sub-groups, i.e., accessions released before 1951, 1951–1960, 1961–1970, 1971–1980, 1981–1990, and 1991–2001. The frequency of *Hap*-2-1A continuously increased, suggesting progressive selection (Figure 6).

## 4. Discussion

Knowledge-based genetic improvement of crop plants is crucial for global food security in the future [21]. Molecular plant breeding uses phenotypic and genetic variations with contemporary tools to develop new varieties with higher productivity and that are resilient to extreme environments [37].

The EcoTILLING approach has been used to discover natural genetic variation in different crop plants such as *Arabidopsis*, wheat, barley, *Brassica* sp., and rice [20,38,39]. The *SSIV* gene has been characterized in *Arabidopsis*, rice, and wheat; however, its allelic variations were not characterized systematically. Our primary goal was to identify natural genetic variation of *TaSSIV* in wheat and its association with traits contributing to yield. EcoTILLING successfully identified 38 SNPs across three homeologous *TaSSIV* genes in modern cultivars and landraces from China and Pakistan.

Development of high-throughput molecular markers is of utmost importance for use in wheat molecular breeding [40]. Gel-free KASP assays could significantly improve the speed and efficiency of selection in wheat breeding programs. Although EcoTILLING successfully identified all the allelic variation in *TaSSIV* homeologous genes, it is a laborious and costly method for routine genotyping. To overcome this problem, KASP markers were developed for several SNPs for high-throughput and cost-effective genotyping of these variants. The six KASP assays developed in our study can be routinely used for identification of SNPs in *TaSSIV* in other germplasm and breeding populations with extremely high accuracy [41].

The allelic variation and KASP markers developed to distinguish *TaSSIV* haplotypes can be instrumental in MAS, which can be utilized in combination with other FMs for TGW and SPL breeding. Wheat A and B sub-genomes have a relatively broader genetic base compared to the D sub-genome. Additionally, non-coding regions also have relatively higher levels of polymorphism compared to coding regions [42]. The absence of significant polymorphism in *TaSSIV-D* is probably due to the narrow genetic background of the D sub-genome or allele fixation due to an evolution and domestication bottleneck.

A haplotype block combining two or more SNPs in strong linkage disequilibrium are more explanatory than bi-allelic SNPs [43]. Beyond bi-allelic SNP variations, haplotype data can capture associations that evade identification by single SNPs [44]. Haplotype-based analyses are still rare in wheat with few exceptions [45,46]. Identification of haplotypes can also capture epistatic interactions between SNPs. Hence haplotype-based approach could increase prediction accuracies [47].

The starch synthase family in plants encodes many starch synthesis genes that play essential roles in starch synthesis and ultimately increase yield [48]. There is a strong relationship between starch and grain yield in wheat and maize. Association analysis is a powerful approach to investigate marker trait associations (MTAs) [49]. In the present study, two haplotypes of *TaSSIV* (*Hap-2-1A* in *TaSSIV-A* and *Hap-3-1B* in *TASSIV-B*) were significantly (*p* < 0.05) associated with yield-related traits in wheat.

It has been demonstrated that grain yield in France [50], Italy [51], the UK, and China [52] has largely been attained by improvements in grain number per square meter with significant change in individual grain weight [53,54]. Accessions possessing haplotype *Hap-2-1A* had higher TGW while accessions having *Hap-3-1B* had larger SPL. Yield-related traits of cereal crops are governed by multiple genes and are influenced by environmental factors [55]. Among the reported gene determinants of cereal grain traits, some genes act constitutively under different environments, while others function in specific conditions [56]. In this study, yield-related traits showed significant association with the identified haplotypes, which suggests that use of these haplotypes can be instrumental to improve yield of the wheat crop.

In China, the geographical distribution of two haplotypes is also supportive. Varieties bred in regions I to IV usually have bigger grain size and larger spikes, but fewer tillers to evade canopy structures favoring rust epidemics [57]. Region II covers ~40% of the total national wheat area and accounts for ~45% of total wheat production in China [36]. Average TGW of varieties in this region is 42–43 g. From landraces to modern cultivars, positive selection of haplotype *Hap-2-1A* favored wheat breeding. Regions I, II, and III account for about 64.8% of total national wheat area and greater turnover of wheat cultivars was also reported from these regions [35]. In China, wheat yield increase has largely depended on higher TGW [55]. There was a significant association between haplotype *TaSSIV-1A*-2 and TGW in MCC (Figure 3). In Northern China, 1.3% genetic gain for TGW has been achieved yearly for three decades with concurrent increases in grains per spike in Yellow and Huai River Valleys winter wheat region [52]. Improvement in TGW and grain weight per spike has also been reported in the Southern China winter wheat region since 1949 [52].

Choice of populations plays a critical role to detect MTAs via association analysis. Accessions from Pakistan were also used to evaluate the *TaSSIV-A* haplotypes in five different geographical regions. In these accessions, the favored haplotype (*Hap-2-1A*) was selected in major wheat growing regions of Pakistan especially in Punjab regions (rainfed region and irrigated region) (Figure 5). In both populations (China and Pakistan) the frequency of the favored haplotype (*Hap-2-1A*) was reasonably high, indicating the positive selection during modern wheat breeding. Although both populations have different population structures, unconscious selection of the favored haplotype is likely due to the high linkage disequilibrium of major yield-related genes selected during selection breeding. However, the frequency analysis in wheat cultivars from other regions like Europe, USA, and Australia would be required to draw the conclusion that selection of favored haplotypes of *TaSSIV* is likely due to the selection of major yield-related genes [35].

In conclusion, we have demonstrated that EcoTILLING is an efficient approach for allele mining of wheat candidate genes and KASP is a robust approach to validate SNPs for further analysis. This study identified 38 unique alleles in *TaSSIV* among 362 wheat accessions collected. Association analysis showed that two haplotypes in *TaSSIV* were significantly associated with TGW and SPL and these haplotypes can be used in future studies to assess their usefulness as selection criteria for improving these yield-related traits.

## Figures and Tables

**Table 1:** List of nucleotide polymorphism in *TaSSIV* with their effects on codons and variation in amino acid.

No.	Gene	Genotype	Allele	Mutation Type	Variation in Amino Acid
1	*TaSSIV-A*	Laoqiaomai	A1447G		Non-coding
2		Sankecun	T1455G		Non-coding
3		Wenmai-6	C1563T		Non-coding
4		Yangmai-158	A1673T		Non-coding
5		Yangmai-158	C2436T	Missense	R77C
6		Guinong-10	T2073G		Non-coding
6		Zhongzhou-741	A2403C	Missense	I66L
8		Mahuaban	G5081A		Intron
9		Huoliyan	C5952T		Intron
10		Huangshuibai	T6013:		Intron
11		Guinong-11	G2143A		Non-coding
12	*TaSSIV-B*	Dingxingzhai	C1529T	Missense	S149L
13		Pingyuan-50	C1560T	Silent	F159=
14		Dabaimai	A2234G	Missense	K384R
15		Lumai-1	T3314G		Intron
16		Lumai-1	T3325G		Intron
17		Xinmai-18	A3478ATT		Intron
18		Baipixiaomai	C6107T	Silent	I822=
19		Xiaobaimai	G6329A		Intron
20		Shahkar-95	A2950:		Intron
21		Drawar-97	A2991G		Intron
22		Blue silver	C3457T		Intron
23		Lasani	G4115CTT		Intron
24		Kohistan	C4176T		Intron
25		Marvi	T3268G		Intron
26	*TaSSIV-D*	Zijuhong	T5904:		Intron
27		Hongchumai	A6168C		Intron
28		Hongmai	G6485:		Intron
29		Hongmai	T6440C		Intron
30		Kabka-3	A3387:		Intron
31		Chumai	C3593G	Silent	G454=
32		Yangmai	C3899G	Silent	V505=
33		Hongpidongmai	T3981A	Missense	S533T
34		FSD-08	C5849T		Intron
35		BWP-6309	G6164T		Intron
36		Manthar	G6070A	Missense	V800I
37		Kohistan-97	A6540G	Missense	T841A
38		Sehar	T4016C	Silent	Y533=

Note: only one accession was listed as a representative due to limited space. “:” Deletion; “=” No change in amino acid.

**Table 2:** Nucleotide polymorphisms and functional marker development of *TaSSIV-A* and *TaSSIV-B*.

Gene	Haplotype	Position of Nucleotides				Gene	Haplotype	Position of Nucleotides	
*TaSSIV-A*		1673 nt	2403 nt	2436 nt	5952 nt	*TaSSIV-B*		1560 nt	6107 nt
	*Hap-1-1A*	A	A	T	C		*Hap-1-1B*	C	C
	*Hap-2-1A*	A	C	T	T		*Hap-2-1B*	T	C
	*Hap-3-1A*	A	C	T	C		*Hap-3-1B*	C	T

## Supplementary Materials

The following are available online at https://www.mdpi.com/2073-4425/10/4/307/s1, Table S1: List of modern and land races accessions of China (MCC) and Pakistan, Table S2: List of primers used for EcoTILLING of TaSSIV A, B and D sub genomes, Table S3: KASP markers that are tightly linked to TaSSIV-A and TaSSIV-B and their primer sequences, Figure S1: Specific primer for TaSSIV-A and TaSSIV-B with validation Chinese Spring, nullisomic, tetrasomic lines.
