# Supplementary material for: EcoTILLING Reveals Natural Allelic Variations in Starch Synthesis Key Gene TaSSIV and Its Haplotypes Associated with Higher Thousand Grain Weight
Source: Genes (Basel). 2019 Apr 18;10(4):307. doi: 10.3390/genes10040307 (PMC6523294; doi:10.3390/genes10040307)
Supplement: Supplementary file 1 [file genes-10-00307-s001.zip › genes-457579-SI/Supplementary files Table S2, S3 and Figure S1.pdf]

Table S2. List of primers used for EcoTILLING of *TaSSIV A, B* and *D* sub genomes.

Primer	Sequence (5'- 3')	Size	Temp
**SS4b-FA1**	TTTTCGTGTAGGTCCAGCAA	20	57
**SS4b-RA1**	CCTTTTATGAACCATTTCTCGC	22	
**SS4b-FA2**	TTTCCATTTCTTGTGTGCTG	20	56
**SS4b-RA2**	AACACTCGGTTGGTTCACTG	20	
**SS4b-FA3**	CCCTTTGACATTTAACCTTCAG	22	56
**SS4b-RA3**	CCGATAAATGTGATGACAAACG	22	
**SS4b-FB1**	TGCATAACCACTGAATTGCA	20	57
**SS4b-RB1**	TTAAGGCTACATCCATTCACAC	22	
**SS4b-FB2**	GTGTGAATGGATGTAGCCTTA	21	52
**SS4b-RB2**	GCCAAAAAAGTCTACATCG	19	
**SS4b-FB3**	TGGCTTCTTCCATACTTTGATAG	22	57
**SS4b-RB3**	GCGCATACTACGATCTTGAGG	21	
**SS4b-FD1**	GGTAGGAATGATACAGAACACC	22	57
**SS4b-RD1**	ACTAAAACCCACTTTGCGAC	20	
**SS4b-FD2**	CTGCAAAAAAATTGTCTAAAAGCTAC	25	58
**SS4b-RD3**	CATGCTTTGAAATTATCTACTTTCG	25	
**SS4b-FD3**	ACCAGAAATTCAGGTGCGTT	20	60
**SS4b-RD3**	TGAGTCGTGTTGTGCCCG	18	

Figure S1. Specific primer for *TaSSIV-A* and *TaSSIV-B* with validation Chinese Spring nullisomic-tetrasomic lines.

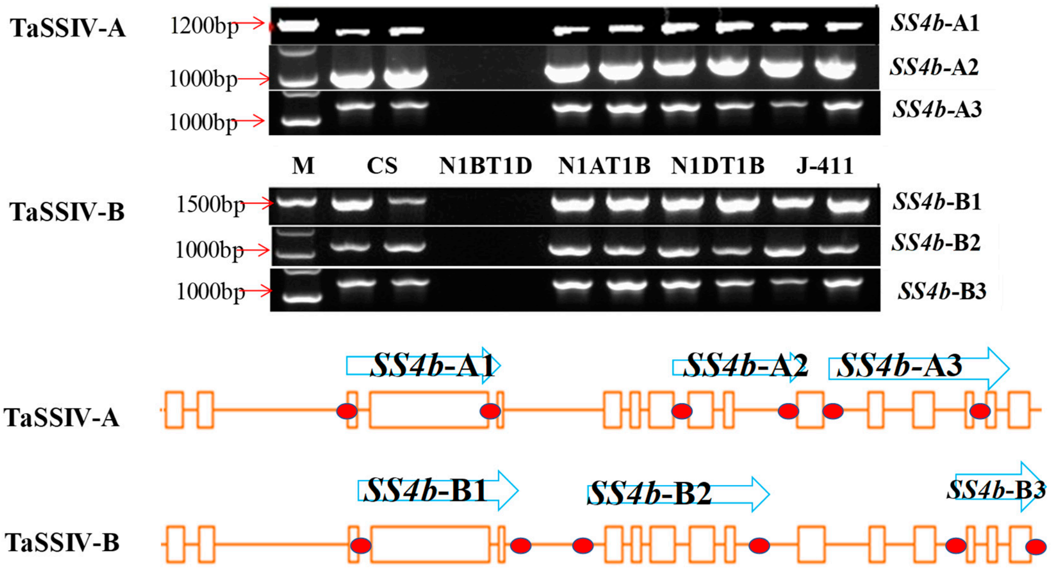

a. Chinese Spring (CS), nullisomic-tetrasomic lines (N1AT1B, N1AT1D, N1BT1D and N1DT1B) and wild type (J411) amplified. b Diagram of *TaSSIV-A* and *TaSSIV-B* primers: orange arrowheads indicate exon regions; blue rectangles represent the location of each primer.

Table S3. KASP markers that are tightly linked to TaSSIV-A and TaSSIV-B and their primer sequences.

**Marker name**	**Sequence (5'- 3'）**
KASP-A1673T	F: GAAGGTGACCAAGTTCATGCTCAAAAAGTCAAATTGGTAGAAGAAA<br>F: GAAGGTCGGAGTCAACGGATTCAAAAAGTCAAATTGGTAGAAGAAT<br>R: TGAGAATGCATTTCACAATTACATG
KASP-A2403C	F: GAAGGTGACCAAGTTCATGCTAAGTTCTCTTGTTCTCTTGTTGATA<br>F: GAAGGTCGGAGTCAACGGATTAAGTTCTCTTGTTCTCTTGTTGATC<br>R: CTTCTACCAATTTGACTTTTTGCTG
KASP-*C2436T*	F: GAAGGTGACCAAGTTCATGCTGAATTTCACGATACTCTTAGCAAAC<br>F: GAAGGTCGGAGTCAACGGATTGAATTTCACGATACTCTTAGCAAAT<br>R: CCAGTGATCTTTTCTCACTTTCCTC
KASP-C5952T	F: GAAGGTGACCAAGTTCATGCTCCTCATGAACAGTTTATTTATCACC<br>F: GAAGGTCGGAGTCAACGGATTCCTCATGAACAGTTTATTTATCACT<br>R: AATACACCATTCCAGACTCATGTTC
KASP-C1560T	F: GAAGGTGACCAAGTTCATGCTTAAGCTTTCAAGTGAAGGGAATTTC<br>F: GAAGGTCGGAGTCAACGGATTTAAGCTTTCAAGTGAAGGGAATTTT<br>R: ATCAAACTCTAATGCTAGTGGAGAG
KASP-C6107T	F: GAAGGTGACCAAGTTCATGCTCATGAGATATGGTTCTGTGCCAATC<br>F: GAAGGTCGGAGTCAACGGATTCATGAGATATGGTTCTGTGCCAATT<br>R: CTGTCATTCAGCCCACCAGTTTTCC
